# Noncontact atomic force microscopy II

**DOI:** 10.3762/bjnano.5.31

**Published:** 2014-03-12

**Authors:** Mehmet Z Baykara, Udo D Schwarz

**Affiliations:** 1 Department of Mechanical Engineering and UNAM-Institute of Materials Science and Nanotechnology, Bilkent University, Ankara 06800, Turkey; 2 Departments of Mechanical Engineering & Materials Science and Chemical & Environmental Engineering and Center for Research on Interface Structures and Phenomena (CRISP), Yale University, P.O. Box 208284, New Haven, CT 06520-8284, USA

**Keywords:** atomic force microscopy, scanning force microscopy

In order to visualize the atomic structure of materials in real space, a microscope with sub-nanometer resolution is needed. As such, breaking the resolution limit associated with the wavelength of visible light employed in traditional optical microscopy has been a long-standing dream of scientists around the world. This goal was finally reached in the early 1980s with the invention of the scanning tunneling microscope (STM). While it is possible to obtain atomic-resolution images of material surfaces by using STM with relative ease, its basic operational principle depends on the phenomenon of *quantum tunneling*, rendering the technique applicable only to conductive and semi-conductive samples. The atomic force microscope (AFM), which was invented only a few years after the introduction of the STM, overcame this fundamental limitation and was used with great success to image a number of sample surfaces with nanometer resolution without limitations associated with electrical conductivity. However, unlike the STM, the operation of the AFM in its traditional form requires the establishment of a permanent – albeit *light* – contact between the probe tip and the sample surface, leading to a finite contact area, which prevents true atomic-resolution imaging.

True atomic resolution imaging through AFM was finally achieved in 1994 with the invention of *noncontact atomic force microscopy* (NC-AFM). The basic idea behind NC-AFM is based on the detection of minor changes in the resonance frequency of a micro-machined cantilever carrying a sharp probe tip due to attractive force interactions while it is oscillated above the sample surface to be investigated. Since actual contact with the sample is avoided, the probe tip retains its sharpness and atomic-resolution images may be obtained. Since its introduction two decades ago, NC-AFM has indeed been used to image a large number of conducting, semi-conducting, and insulating material surfaces of technological and scientific importance with atomic resolution, thus contributing to nano-scale science in a major way with each passing year. The capabilities of NC-AFM are not only limited to atomic-resolution imaging: Force spectroscopy allows characterization of interatomic forces with unprecedented resolution in three spatial dimensions, while manipulation experiments at both low temperatures and room temperature have demonstrated the capability of the technique to controllably construct atomic-scale structures on surfaces.

While initially small, the NC-AFM community gradually grew with each passing year. To provide a forum for exchange between researchers, progress in the field has been discussed since 1998 at annual conferences held in various cities around the world. The latest meeting in that series, the *16^th^ International Conference on Non-Contact Atomic Force Microscopy* hosted by the University of Maryland in August 2013, demonstrated once again rapid progress in the field. For this Thematic Series, many of the leading groups have provided contributions with the goal of assembling a collection of papers that provide an overview of the current state-of-the-art in NC-AFM research, thereby delivering a snapshot of the newest trends in the field. For example, the realization that experimental results in NC-AFM are often strongly influenced by the mechanical and chemical properties of probe tips have sparked an increase in simulation work aimed at uncovering the associated principles, which is reflected in a number of contributions. Additionally, three-dimensional force spectroscopy on adsorbed molecules as well as challenges associated with the correct incorporation of long-range forces in such experiments are emphasized. Finally, it becomes apparent how new experimental methods that are based on the working principle of NC-AFM are continuously being developed, which is documented by a number of papers dealing with multi-frequency AFM as well as Kelvin probe force microscopy (KPFM).

We thank all the scientists who have submitted their outstanding work to the second edition of this Thematic Series, the referees for their careful reviews, and the *Beilstein Journal of Nanotechnology* for providing a truly open-access forum for publication and dissemination of research results. We hope that the papers presented here will contribute their share to stimulate new ideas and inspire new directions for future research.

Mehmet Z. Baykara and Udo D. Schwarz

Ankara, New Haven, February 2014

